# Burn trauma from a space heater requiring paraspinal muscle flap back reconstruction with skin grafting: A case report

**DOI:** 10.1016/j.tcr.2022.100738

**Published:** 2022-11-23

**Authors:** Katherine Stuart, John Culhane, Dan Brown, Philippe Mercier, Sumesh Kaswan, Heidi Israel, Vicki Moran

**Affiliations:** a Department of Surgery, Saint Louis University School of Medicine, St. Louis, MO, USA; b Department of Trauma Surgery, Sanit Louis University School of Medicine, St. Louis, MO, USA; c Division of Surgical Critical Care, Saint Louis University School of Medicine, St. Louis, MO, USA; d Division of Neurological Surgery, Saint Louis University School of Medicine, St. Louis, MO, USA; e Division of Plastic and Reconstructive Surgery, Saint Louis University School of Medicine, St. Louis, MO, USA; f Division of Orthopedic Surgery, Saint Louis University School of Medicine, St. Louis, MO, USA; g SSM Health Saint Louis University Hospital, St. Louis, MO, USA; h Saint Louis University School of Medicine, St. Louis, MO, USA

**Keywords:** Full-thickness burns, Multidisciplinary team, Wound care, Skin graft

## Abstract

Full-thickness burns damage all layers of skin and may also damage underlying tissue including bones, muscles, and tendons. Full-thickness burns almost always require immediate medical and surgical management. Some may require extensive bone, muscular, and other reconstructive surgery depending on the depth of involvement of surrounding tissues. Bone exposure in burn patients can lead to unique complications including osteomyelitis. We present the case of an elderly patient with a history of dementia who presented with full-thickness burns to the back with exposed spinal elements who later developed osteomyelitis requiring lumbar spine reconstruction with bilateral paraspinous muscle flap for back reconstruction, adjacent tissue transfer, and split thickness skin grafting. This case represents the severity of full-thickness burns with underlying bone exposure and the importance of aggressive wound care and multidisciplinary team approach.

## Case report

**Fig. 1:**
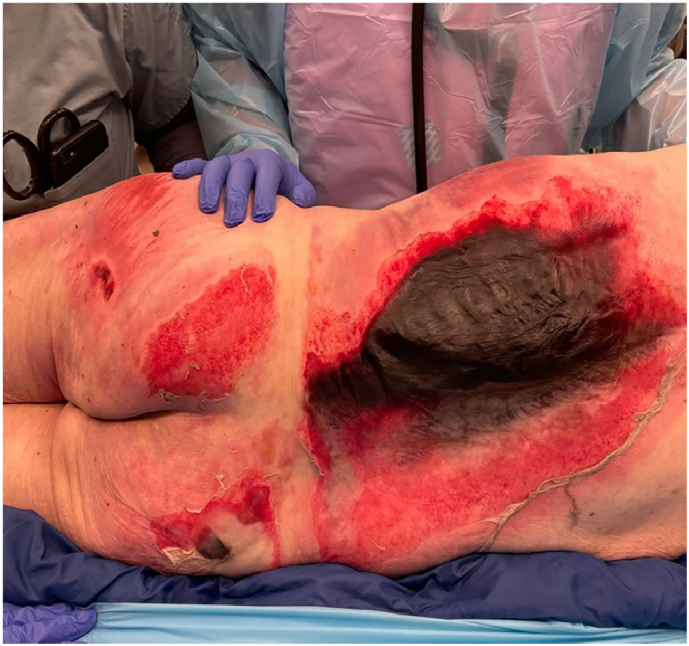
Burns to back and buttock noted upon admission on 2/20/21.

The patient was a 78-year-old male with a past medical history of diabetes, brought in by EMS after reportedly sleeping on a space heater outside his home on 2/20/21 causing partial and full-thickness burns to his back and buttock. The patient arrived with an intact airway, GCS 15, and no other evidence of trauma other than the burns to his back and buttock which were measured as 9 % full-thickness eschar from mid-spine to left flank with surrounding partial thickness and superficial burns and 4–5 % partial thickness burns to bilateral buttocks with scattered areas of full-thickness burns. He was admitted to the Trauma ICU and aggressive fluid resuscitation and wound care were initiated (Fig. 1).

He was taken to the OR on 2/22/21 for excisional debridement which resulted in wounds measuring 9 cm × 10 cm × 1 cm and 2 cm × 7 cm × 2 mm to the back and buttock. Areas of healthy subcutaneous fat were noted at the superior portion of the wound with nonviable muscle overlying the spinous process. A negative pressure wound therapy device (NPWT) was applied.

He was next taken to OR on 3/1 where areas of nonviable skin and muscle were noted requiring debridement to the subcutaneous layer on the bilateral buttocks. Upon completion of the procedure, the wounds measured: back 30 × 21 × 1 cm, right buttock 9 × 8 × 1 cm, and left buttock 4 × 5 × 1 cm. A NPWT was placed to the back and right buttock wounds. Mepilex dressing was applied to the left buttock.

**Fig. 2:**
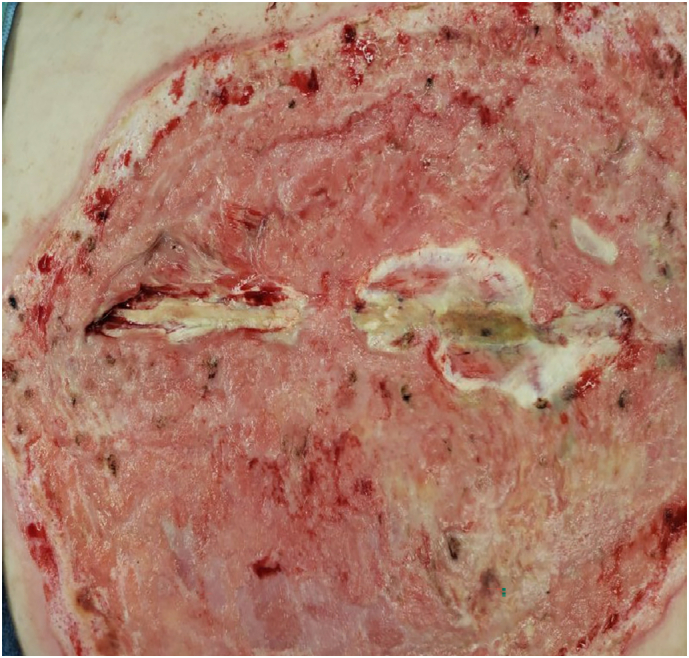
Post-debridement of back wound (3/5/21).

Additional debridement and split thickness skin grafting was performed in OR on 3/5 (Fig. 2). Skin grafts were taken from bilateral posterior thighs and placed on the back and right buttock burns. NPWT was applied over the 800 cm^2^ grafted wound.

**Fig. 3:**
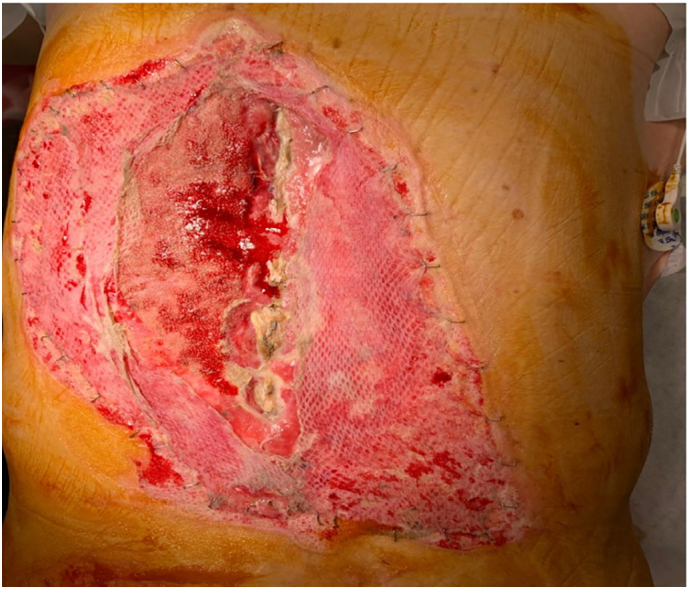
Pre-op: Before mucle flap closure (3/11/21).

The patient returned to OR on 3/11 with Plastics and Reconstructive surgery, Trauma Surgery, and Neurosurgery (Fig. 3). The back wound at that time measured 30 × 8 × 2 cm. The patient underwent thoracic and lumbar spine reconstruction with successful resection of spinous processes and closure of spinal wound which included the following: right and left paraspinous muscle flap for back reconstruction as well as adjacent tissue transfer at the back defect following bilateral paraspinous muscle flap harvest for a total area of 250 cm × 2 in the mid back area (25 × 5 cm on the right side and 25 × 5 cm on the left side). A split thickness skin graft over the bilateral paraspinous muscle flaps for an area of 25 × 8 cm was also performed. The area over the lower back graft site measuring 25 × 8 cm was covered with NPWT.

The patient tolerated a total of 4 operations and required 4 units of PRBCs during his clinical course. He was transferred out of ICU to Trauma floor service on 2/23 and was discharged to a rehabilitation facility on 3/18. The patient's clinical course was complicated by a decreasing oral intake during admission likely secondary to his underlying dementia. A Dobhoff tube was placed in OR on 3/5 to ensure adequate nutrition. The Dobhoff was not tolerated and after several self-removals and reinsertions by floor team, the tube was not replaced following final self-removal on 3/7. Due to persistent nursing care, the patient was able to tolerate eating partial meals and meal supplements during the remainder of his clinical course.

**Fig. 4:**
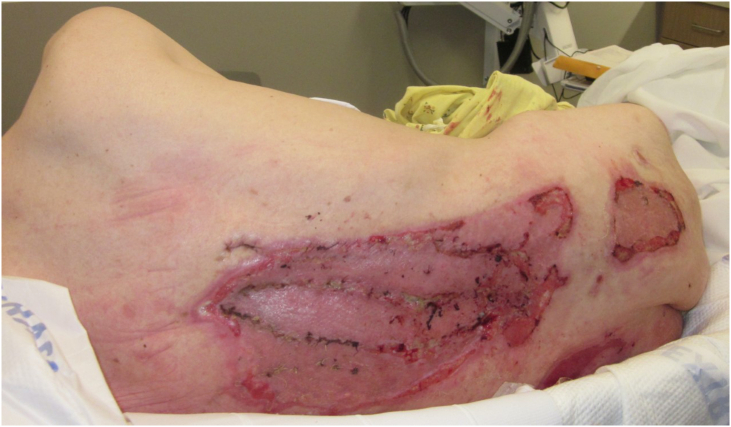
3 Weeks post-split-thickness skin grafting with muscle flap (3/31/21).

After the second debridement on 3/1, the patient's WBC began to trend upward. Given the exposed bone in the patient's wound, there was concern of developing osteomyelitis. Wound cultures were obtained on 3/5 in OR as well as blood and fungal cultures. Cefepime and vancomycin were started empirically. Blood cultures grew *Fusobacterium nucleatum*. Wound cultures grew: *Pseudomonas aeruginosa*, *Streptococcus agalactiae* (GBS), *Enterococcus faecalis*, Enterococcus cloacae, and *Staphylococcus aureus*. The Infectious Disease Service was consulted and agreed with the osteomyelitis diagnosis. They recommended continuation of cefepime and vancomycin, and addition of metronidazole to complete a 6-week antibiotic course from last OR debridement. A PICC line was placed on 3/18 for further intravenous antibiotics before discharge to the rehabilitation facility. The patient was seen in outpatient clinic three weeks after discharge which showed intact skin grafts with healthy granulation tissue present below dressings (Fig. 4).

## Discussion

Historically, there have been several systems of classifying burns. The medical field now largely uses a thickness approach for classifying burns broken down into superficial, partial, or full-thickness burns. Using this convention, fourth degree burns constitute full-thickness burns [1]. These burns commonly form eschar which is collections of dead tissue and dried secretions from the skin wound that provide a temporary coverage and often slough off independently [2]. In both partial and full-thickness burns, the epidermis as well as the immune protection it offers is lost leaving these patients vulnerable to infection, fluid loss, and temperature instability.

In recent years, negative pressure wound therapy (NPWT) has been used as a barrier for infection prevention and a means of creating ideal conditions for healing, which are believed to work by stimulating angiogenesis and mitogenesis as well as evacuating fluid resulted in well-perfused granulation tissue that often allows for a simpler reconstruction option [3]. The reconstructive ladder in burns therapy is usually as follows: primary closure, skin graft, local flap, regional flap, distance flap, and finally free flap for those burns where vital structures are exposed [4].

For this patient, after initial debridement, the spinous process of several lumbar vertebrae and underlying neuro bundle were exposed. Given the increase risk of osteomyelitis as well as meningitis, neurosurgery services, orthopedic spine services, and infectious disease services were consulted. In an attempt to prevent the possible complications of these injuries, after the initial debridement and escharatomy, NPWT was immediately implemented. Though the patient did ultimately develop osteomyelitis, the NPWT did serve its purpose of promoting healing and the formation of granulation tissue noted at each OR return.

For burn patients, special attention should be given to increased nutritional needs given the hypermetabolic response to trauma which has led to continuous enteral feeding commonly becoming standard of care [5]. Nutrition proved to be difficult given the patient's underlying dementia leading to poor appetite and repetitive pulling of Dobhoff feeding tube. Through nursing staff efforts and Nutritionist recommendations, the patient lost only 2 kg during hospitalization highlighting the importance of adequate nutrition to promote the best chances of healing.

## Conflict of interest

None.
